# Supplementary figures and images for: Targeted DamID detects cell-type-specific histone modifications in intact tissues or organisms
Source: PLoS Biol. 2025 Mar 11;23(3):e3002944. doi: 10.1371/journal.pbio.3002944 (PMC12135883; doi:10.1371/journal.pbio.3002944)

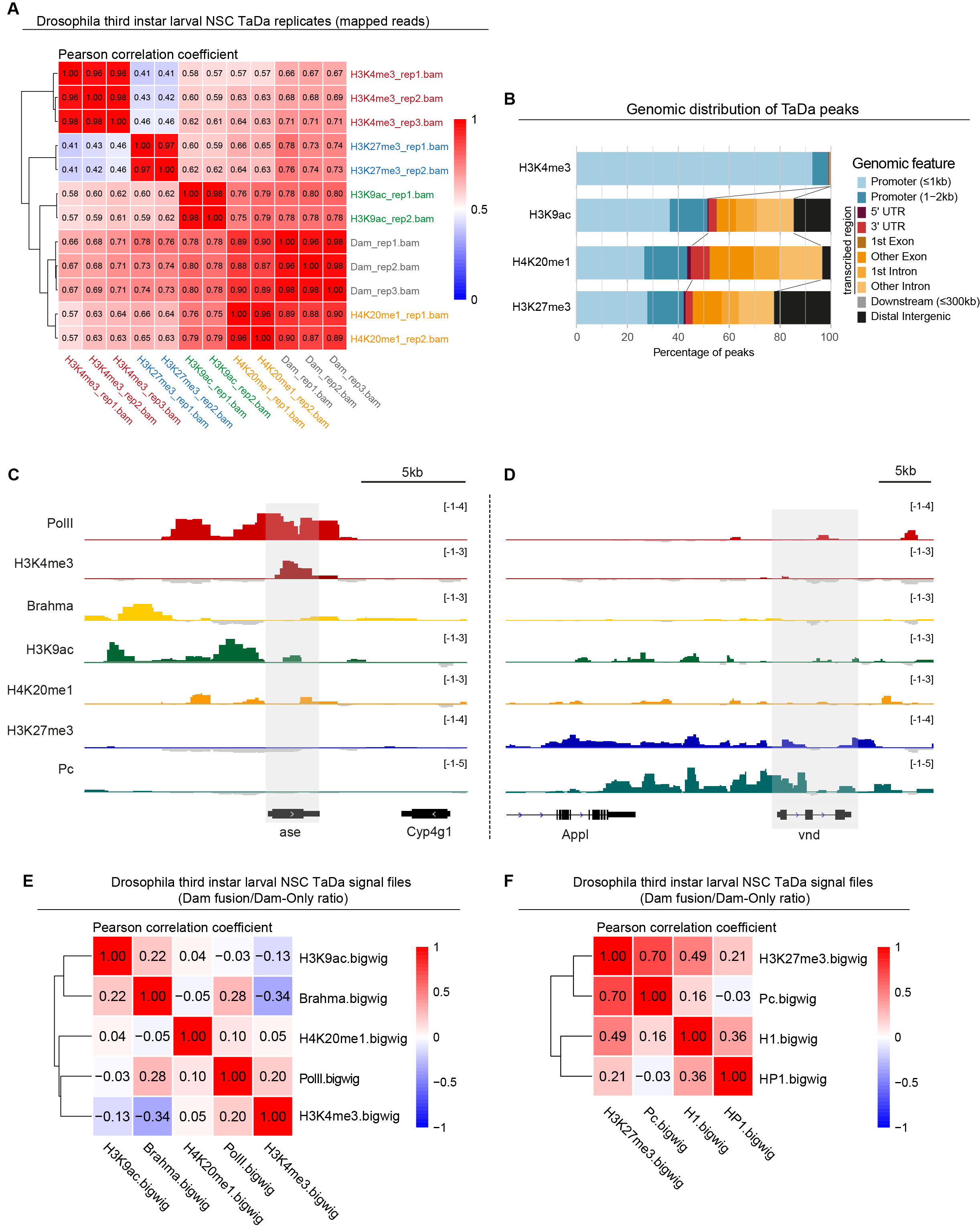

Supplement: S1 Fig — (A) Pearson correlation coefficients of aligned reads between all TaDa replicates from Drosophila third-instar larval NSCs and Dam-only conditions. (B) Genomic feature distribution of TaDa profiles in Drosophila third-instar larval NSCs. (C) TaDa profiles in Drosophila third-instar larval NSCs at the asense locus (shaded). (D) TaDa profiles in Drosophila third-instar larval NSCs at the vnd locus (shaded). (E–F) Pearson correlation coefficients of normalized and averaged signal files (bigwig) between TaDa using chromatin readers and previously profiled chromatin-binding proteins (Marshall and Brand, 2017). All sequencing files are available at GSE278272 or referenced publication. (TIF) [file pbio.3002944.s001.tif]

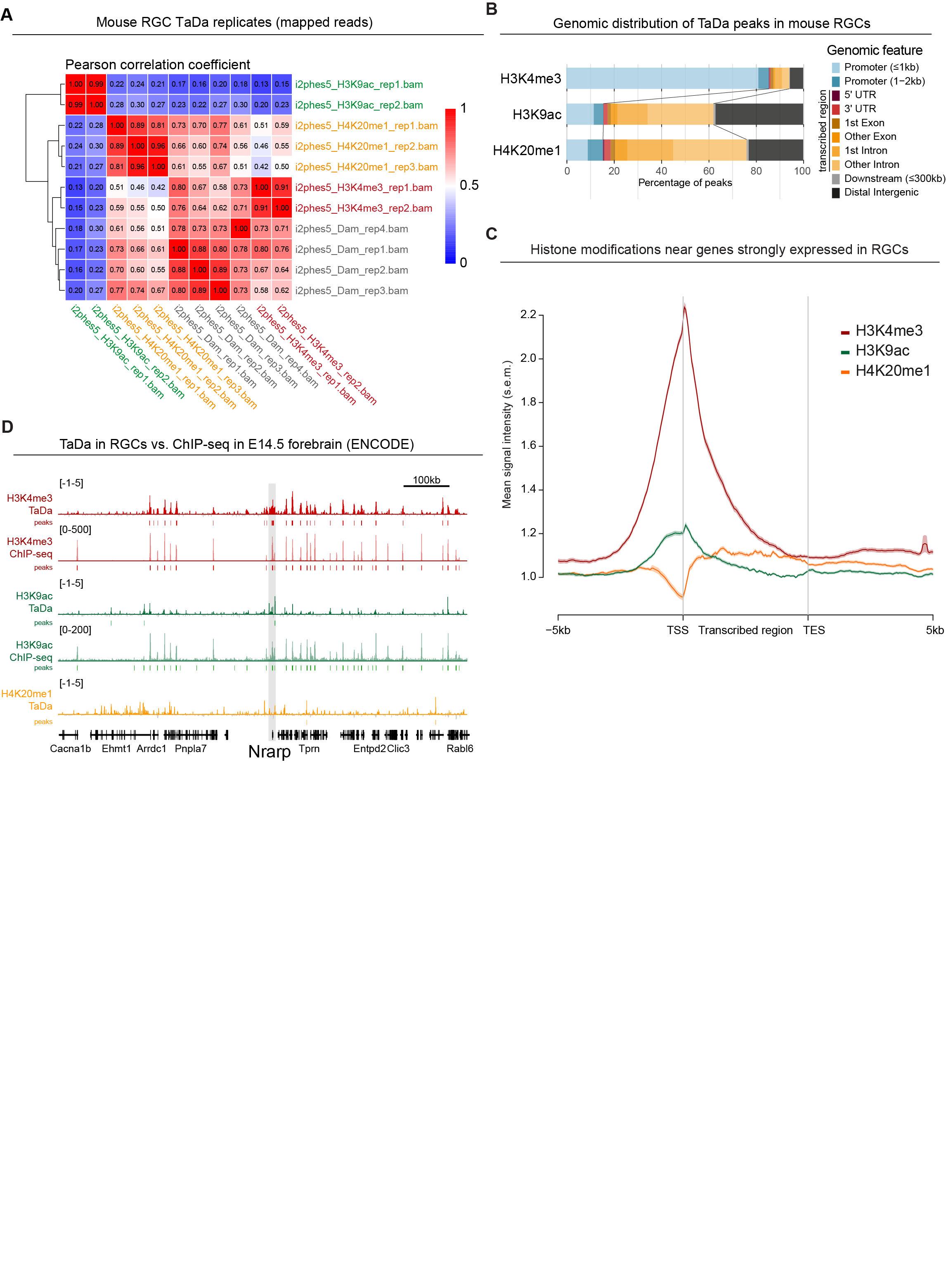

Supplement: S2 Fig — (A) Pearson correlation coefficients of aligned reads between all mouse RGC TaDa replicates and the Dam-only conditions. (B) Genomic feature distribution of histone mark TaDa profiles in mouse RGCs. (C) Average signal intensity (±s.e.m.) of TaDa signal at genes (TSS to TES ±5 kb) expressed in mouse RGCs. Signal files in bigwig format are available as supplementary at GSE278272, with the following filename prefixes: H4K20me1: GSE278272_iue115_5_phes5_15f11nls_5-vs-iue60_i2phes5_dam_S6, H3K4me3: GSE278272_iue62_phes5_taf3-vs-iue53_i2phes5_dam_S2, H3K9ac: GSE278272_iue92_i2phes5_19e5-vs-iue61_i2phes5_dam_S1. (D) TaDa (top) and ChIP-seq (ENCODE) profiles for histone modifications near the Nrarp genomic locus (shaded). All sequencing files are available at GSE278272. (TIF) [file pbio.3002944.s002.tif]
