# Supplementary material for: Targeted DamID detects cell-type-specific histone modifications in intact tissues or organisms
Source: PLoS Biol. 2025 Mar 11;23(3):e3002944. doi: 10.1371/journal.pbio.3002944 (PMC12135883; doi:10.1371/journal.pbio.3002944)
Supplement: Attachment — Submitted filename: Response to reviewers.pdf [file pbio.3002944.s005.pdf]

Reviewer #1: In their study, van den Ameele et al. expand their previously developed TaDa technique to profile histone modifications genome-wide, in a cell-type specific manner. The great tool developed by the authors allows to track histone mark patterns in cell types in which conventional approaches like ChIP-seq or DamID-seq are not easily performed. They probe the pattern of several histone marks: H3K4me3, H3K27me3, H3K9Ac and H4K20me1; in different biological systems: drosophila neural stem cells, mouse radial glial cells and xenopus embryos. This approach will enable profiling of histone marks or other chromatin factors in a wide range of tissues or cell types. However, this manuscript would benefit from several clarifications, especially about data analysis and data visualization.

My comments to improve this manuscript are:

Major points:
1) Figure legends should be more specific and describe in more details the corresponding panels. Some key information seems missing, such as:
  a. A description of the clustering done in Figure 1F.

The localisation of the histone marks around gene features is more clearly represented in Figure 1E (now 1F). We have therefore removed Figure 1F.

  b. A description of the color code in Figure S2E. Related to that panel,
    i. How is the NES calculated?
    ii. What do the arrows represent?

The analysis of normalised enrichment scores was initially included to show similarity of histone modification profiles acquired by DamID with data from the ENCODE consortium database. However, we do agree with the reviewer that this analysis is confusing and perhaps redundant, as it compares these marks in very different contexts (e.g. cell types). We show a comparison of the histone marks attained using DamID and ChIP between concordant cell types in Figure S2D and the analysis involving the normalised enrichment score has been removed from the supplemental data.

2) To further show the specificity of their H3K4me3 TaDa signal in Xenopus embryo, the authors may want to correlate their data obtained in the endoderm with those previously obtained by ChIP-seq in the same cells (Hörmanseder et al., 2017).

We are confident in the specificity of the reader, as demonstrated in our Drosophila and mouse data.

3) What is the temporal resolution of TaDa? Could H3K4me3 pattern be assessed at earlier time points in Xenopus development? What is the author's opinion on this?

DamID could be used to assess H3K4me3 at earlier time points in Xenopus development, although we have not tested this as part of the present study.

4) Have the authors mapped H3K27me3 in Xenopus embryos? Performing such an experiment may further consolidate the feasibility of their technique in this organism.

We agree with the reviewer that this would be interesting and would further consolidate the use of the tool, although it was not within the scope of the current study.

5) In Figure 1C-D and Figure S1C-D, the TaDa signal depicted on IGV tracks ranges from of 0 to 3. However, in Figure1E, the y-axis representing "the average signal intensity of TaDa signal across genes" ranges from -0.2 to 0.2. Could the author explain why there is such a difference between these two scales? How can H3K27me3 signal have negative values? A similar plot can be found in Figure S2H, which has a scale ranging from 0 to 2.2.

We thank the reviewer for spotting this error, the scales on Figure 1C-E should start at -1, as the values are a Dam-fusion/Dam-Only ratio, where Dam-Only reads are higher than Dam-fusion the signal is negative. The scale depends on the levels of Dam-Only binding i.e. chromatin accessibility in the control. To clarify this, we have also labelled the y-axis in Figure 1F. We have also edited labels in Figure S2H for clarity.

6) Pearson correlation usually ranges from -1 to 1 (as shown in Figure S1E-F). However, in Figure S1A and S2D the Pearson correlation ranges from 0 to 1 only. Can the author comment on such a scale? For a better visualization of the data, the authors may want to use the same scale and color-code for all heatmaps representing Pearson correlation coefficients.

Some correlation analysis was done using a Pearson correlation, which only ranges from 0 to 1, but other analyses was performed using Spearman correlation, which can include negative values. The Figures have been updated to clearly represent this.

7) Related to the previous point, the correlation coefficients between H3K4me3, H3K9Ac and H4K20me1 are very different between Figure S1A and S1E. For example, Figure S1E shows a negative correlation of -0.13 between H3K9Ac and H3K4me3 while Figure S1A shows a positive correlation of ~0.60 between those same marks. What could be the explanation for that?
Please see previous comment. In Pearson correlation 0.6 represents a similar level of correlation to a Spearman value of -0.13.

8) In the Xenopus TaDa section, the authors claim that they map histone modifications in single embryos (last sentence of the paragraph: "TaDa enabled us to profile H3K4me3 genome-wide in a single embryo"). However, it is specified in the methods (DamID-seq section) that five embryos were pooled for genomic DNA extraction. As a consequence, the TaDa technique does not map histone modifications in single

embryos but rather in a limited number of embryos. The authors need to tone down their conclusion of this section.

We thank the reviewer for noticing this error. As stated in the methods, for Xenopus DamID, 5 injected embryos were pooled for genomic DNA extraction, and one fifth of the genomic DNA was processed for DamID. To account for this we have removed all reference to single embryos from the text.

9) To improve the readability of the paper, the introduction should be extended, including more details about the chromatin marks investigated (e.g. their localization in the genome, their role in gene regulation, etc.).

We have extended the introduction to include more extensive discussion of histone modification localisation and functions in gene regulation.

10) The text does not refer to Figures 2D-E, S2A-D or S2G. Please remove those panels from the figures or cite them in the text and reformat the figures accordingly.

We have edited the text to ensure that all Figures are correctly referenced

Minor points:
1) Figure S1A: a couple of numbers are missing in the matrix

Missing numbers have been added.

2) Figure S2F: the track labels are misaligned with the tracks themselves.

Track labels have been realigned throughout.

3) It is mentioned in the first paragraph of the Results that the chromatin mark H3K36me3 was profiled by TaDa. Those data are missing from the Figures, are those data not reliable (as mentioned for H3K9me3 TaDa signal)? Can the authors clarify this point?

To clarify, as part of this study we also made a tool for profiling H3K36me3. We wish to refer to it in the methods section to make it available for other groups to test if useful for their work. We have edited the text to clarify this.

4) In the Methods, "DamID-seq data processing" section: the order of the species and their corresponding reference genome is not correct.

This has been corrected.

5) For a better comprehension and readability of the manuscript, the authors may want to explain in more details their intronDam and floxDam tools, and their use in the mouse brain.

These tools have been published in our previous papers and diagrams are included to assist the reader. IntronDam is described in Wade et al. (2021) and Van den Ameele et al. (2022). floxDam is described in Van den Ameele et al. (2022). The text mentions further detail about these constructs can be found in these studies.

Reviewer #2: The manuscript "Targeted DamID detects cell-type specific histone modifications in vivo" describes the profiling of multiple histone modifications using the authors' previously published targeted DamID (TaDa) method. The author fuse chromatin binding proteins or single-chain antibodies to the Dam adenine methylase Dam in intact tissues to generate cell-type-specific histone modification profiles. The authors apply their method to neural stem cells in Drosophila, neural stem cells in the developing mouse brain, and the 1-cell stage of Xenopus embryos during gastrulation and neurulation. The authors achieve cell-type specificity using either a cell-type specific promoter for the TaDa construct or injecting in vitro transcribed mRNA of the TaDa construct Xenopus embryos. A group recently used DamID bound to mintbodies to detect histone modifications in vivo (PMID: 35366395) but required single-cell sequencing to investigate histone modifications in a cell-type-specific manner while the present manuscript describes a method for achieving cell-type-specificity without requiring single-cell sequencing.
The ability to profile histone modifications in vivo would be of high use to the chromatin community, especially since TaDa is a popular method for profiling in vivo chromatin proteins in Drosophila, and the current manuscript describes a method for effective use in three model organisms. I have only minor concerns.

1). The introduction, discussion, and methods section mention using the PWWP domain of DNMT3A to profile H3K36me3 and a single-chain antibody to H3K9me3. Yet, data for neither modification appears in the manuscript. Please remove mentions of these histone modifications from the text or show data.

As mentioned in our response to reviewer 1 - We made a tool for profiling H3K36me3. We wish to refer to it in the methods section to make it available for other groups to test if useful for their work. We have edited the text to clarify this. The construct using a single chain antibody to profile H3K9me3 was not included in this paper, we thank the reviewer for noticing the mistake and have removed any reference from the text.

2). There appear to be peak annotations beneath all genome browser tracks in all the figures. Please indicate the peak track in the figure or at least in the legend for the figure.

We have added a label to denote where the peak track is.

3). Please add a label to the y-axis of Figure 1E to indicate the units.

The y-axis is representative of a Dam-fusion/Dam-Only ratio. We have added a label to make this clear.

4). In Figure 1F, there is not designation of what C1-C12 on the heat maps indicate. Please correct.

As discussed in our response to reviewer 1, this figure panel has been removed as mark distributions are more clearly shown elsewhere in the figure.

5). The method for generating the images in Figures 2I and 2K is missing from the Methods section; please correct.

Methods for generating these images are in the section "previously published datasets". For clarity, we have also included a reference in the figure legend.

6). Panels A, B, and C of Figure S2 are not mentioned in the text; please correct.

The text has been edited to refer to these figure panels where relevant.

Reviewer #3: In the submitted manuscript, the authors present an extension and validation of their previously published protocol of cell-type specific targeted DamID. DamID takes advantage of a bacterial methyltransferase that, when directed to specific areas of the genome via fusion partners, will create m6A residues at GATC motifs. In this manuscript, the authors demonstrated a system based on cell-type specific promoters to expression fusion proteins and characterize several chromatin marks within drosophila NSCs, mice radial glia, and xenopus embryos. This modification to the protocol is a logical next step yet is impactful and a significant progression from prior work, and the data presented are exciting, with one notable exception that I think needs to be addressed before publication.

Major (essential) issues:

1. I am unconvinced that the H3K27me3 profiling data presented in the paper is working in mouse cells. The data from the drosophila NSCs (Fig 1C) look as I would expect an H3K27me3 profile to look. The data presented in figure 2D and 2E does not qualitatively look like H3K27me3 profiles, however. Instead, they look like the other presented tracks of activating histone modifications. Especially troubling is the divergence between the tracks presented in figure S2F - in general the TaDa tracks look like the ChIPseq tracks, with the exception of the H3K27me3. Additionally, in the correlation matrix shown in Figure S2D, H3K27me3 has a very high correlation with both H3K4me3, but also with the unfused Dam control. Figure S2H also does not look like figure 1E, and the enriched sets for H3K27me3 in figure S2E are mostly activating marks such as H3K27ac and H3K4me3 rather than mostly H3K27me3 sets. In the text, these data are presented as perhaps evidence of bivalent chromatin, but I think these correlations are too high; to me this suggests the fusions may not be working here, and what is actually being measured in the H3K27me3 is something like accessibility (hence the high correlation with the unfused Dam in figure S2D) rather than interactions with the chromatin state. Given its high correlation with the unfused Dam, it is also

possible that the H3K4me3 is also simply showing accessible chromatin, which would be less noticeable given the expected correlation between active promoters and accessible chromatin. This should also be checked especially given that H3K9ac doesn't show this correlation.

We thank the reviewer for noting that the H3K27me3 data is inconsistent with the expected distribution of a repressive mark and is not adequately explained by the possibility of bivalency. We are confident in the data collected using this fusion in *Drosophila* but not in the mouse cortex. Therefore, we have made the decision to remove these data from the paper. The text and figures have been edited accordingly.

Regarding the H3K4me3 distributions, we do expect localisation to open chromatin due to its function as an active histone modification. We normalise the fusion protein signal to a Dam-only control which represents all open chromatin, therefore the enrichment seen is above what would be expected only from open chromatin.

Minor issues:

1. In figures S1B and S2G, the inclusion of an additional bar depicting the genomic (unenriched) proportion of each of the genomic features, would enhance interpretability.

We thank the reviewer for this comment but believe that this figure clearly conveys genomic feature distribution.

2. In figure S2F, the labels of the tracks are vertically misaligned.

The labels of tracks have been aligned throughout all figures.

3. Given that DamID is resolution-restricted to GATC motifs, the early data processing steps are described as occurring in GATC-bounded bins, later analysis occurs in genomic coordinates. Does it make sense to stay in bin-space rather than genome-space for, for instance, peak calling? The methods mention that peaks are merged when within 1kb, but it seems like the local GATC density could affect peak merging here, if for instance a mid-peak GATC-bounded fragment is too large (in nucleotide width) for accurate illumina cluster generation and is artificially lowly-represented. Stitching over bin distance rather than nucleotide distance may alleviate some of this issue? I don't think this is an issue for any analysis presented within the manuscript, but I would be curious how this would affect, for instance, figure 1F.

We thank the reviewer for this suggestion and agree that it would be interesting to test this. Although not included in this study, we will look into this in the future.

4. What do the clusters in figure 1F represent? It seems like the biggest correlation (for, say, clusters 3,4,6,9,10,11) is with horizontal position of H3K4me3 related to the

TSS? Is there a biological significance there, or is this an artifact of something like local GATC density?

As mentioned in our response to reviewer 1 – Clustering was included to show the localisation of the marks around features of genes, which is more clearly represented already in Figure 1E (now 1F). We have therefore removed this panel.
