# Supplementary material for: Targeted DamID detects cell-type-specific histone modifications in intact tissues or organisms
Source: PLoS Biol. 2025 Mar 11;23(3):e3002944. doi: 10.1371/journal.pbio.3002944 (PMC12135883; doi:10.1371/journal.pbio.3002944)
Supplement: Attachment — Submitted filename: Authors response.docx [file pbio.3002944.s006.docx]

*Reviewer #1:

In their revised version of the manuscript, the authors addressed most of the points raised by the reviewers. However, the authors only partially addressed the major point raised by the editors. Indeed, it was specified in the response to the authors that "reviewer 3's major concern and reviewer 1's point 4 would need to be thoroughly addressed before considering the manuscript for publication", and "more work will be needed to map H3K27me3 in Xenopus and show that the TaDa tracks indeed reflect H3K27me3 detection".

The authors addressed reviewer 3's major comment by removing H3K27me3 data generated in the mouse cortex.

Reviewer 1's point 4 was not addressed, the authors did not map H3K27me3 in Xenopus. Thus, H3K27me3 was mapped in Drosophila neural stem cells only.

**We agree that mapping H3K27me3 in Xenopus would enrich the manuscript, however it is currently out of scope for the study. It was specified in the editors’ response that “However, note that we don't require additional H3K27me3 data”.**

Another important point raised my concern while assessing the authors' rebuttal.
On their answer to reviewer 1's point 6), the author state that: "Pearson correlation ranges from 0 to 1". However, Pearson correlation coefficient, just like Spearman correlation coefficient, ranges from -1 to 1 (ref 1, 2). It is worrisome and I am now unsure of what is actually plotted in Figures S1A and S2A. How do the authors calculate the correlations? Which package are they using? Maybe the authors are in fact plotting the coefficient of determination, R2, which ranges from 0 to 1?
Along a similar line, the authors state in the answer to reviewer 1's point 7) that: "Pearson correlation 0.6 represents a similar level of correlation to a Spearman value of -0.13". Again, this statement is incorrect. The correlation between Pearson and Spearman correlation coefficients depends on the data type (ref 3). For linear and monotonic data, Spearman and Pearson correlation coefficients should be similar.
These points need to be clarified as they question the authors' comprehension of the statistics used in the manuscript.

**We thank the reviewer for noticing this. To ensure analyses were consistent and correct we re-evaluated and repeated all correlation analyses that were performed for the manuscript.**

**Pearson correlation coefficients can indeed range from -1 to 1. To clarify, in this case, the coefficients were all positive, therefore the scale was adjusted. We are confident the coefficients plotted are Pearson correlation coefficients and not coefficient of determination. For these analyses we used deeptools package (v 3.5.1) with multiBamSummary or multiBigwigSummary function, followed by plotCorrelation function with the -pearson flag, as described in Methods.**

**To correct what was stated in the previous response, all correlations performed were, in fact, Pearson correlations (the label of Spearman correlation was incorrect in S1E,F). This was left into the manuscript from a previous version that was since changed. To ensure consistency in these analyses, we repeated all analyses with both Pearson and Spearman correlations for the purpose of this rebuttal (Fig. 1 and 2) and updated the heatmaps in the manuscript.**

**Figure 1. Pearson and Spearman correlations of *Drosophila melanogaster* TaDa reads and signal files, with all scales adjusted to -1 to 1.**

**Figure 2. Pearson and Spearman correlations of Mus musculus TaDa reads, with all scales adjusted to -1 to 1.**

**The text has also been updated to clarify what is being plotted in all figures of the manuscript:**

**In S1A and S2A, Pearson correlation coefficients of raw, aligned reads (bam files) are plotted. The purpose of this comparison is to assess the relationship between the replicates of given datasets. The files, at this point, are not normalised. In S1A and S2A, the scale is adjusted to 0 to 1, with white at 0.5, since all reported coefficients are positive. This was done only for increased readability of the heatmap, and we are aware that the coefficients can be negative, as well.**

**In S1E and S1F, Pearson correlation coefficients of normalised signal files (bigwig files) are plotted. These files are reporting Dam-fusion to Dam-Only normalised ratios over GATC fragments, as calculated by damidseq_pipeline (Marshall and Brand, 2015), averaged among 2 or 3 replicates. The purpose of this comparison is to assess the relationship between the signal of chromatin proteins after normalisation.**

**We also agree that our previous statement of** "Pearson correlation 0.6 represents a similar level of correlation to a Spearman value of -0.13" **was not entirely accurate. As explained above, S1A/S2A and S1E,F use different input files and serve different purposes.**

**When generating signal files, we normalise the Dam-fusion reads to Dam-only reads to create ratios. As we and others reported previously, often the largest contribution to Dam-fusion raw reads is non-specific signal at most accessible GATC sites. As such, the raw reads contain a lot of background signal, which means they are highly correlated to Dam-Only raw reads and each to other** (Marshall and Brand, 2015; Tosti et al., 2018)**. This is especially common for proteins that bind accessible DNA, as Dam is more likely to non-specifically methylate those regions. This is why the correlation coefficients in S1A are different (and often higher) than coefficients of background-normalised signal files in S1E,F.**

**We have repeated all analyses, replotted all correlations, adjusted all relevant labels and Figure legends and edited the methods to reflect the implemented changes.**

**Marshall, O.J., Brand, A.H., 2015. damidseq_pipeline: an automated pipeline for processing DamID sequencing datasets. Bioinformatics 31, 3371–3373. https://doi.org/10.1093/bioinformatics/btv386**

**Tosti, L., Ashmore, J., Tan, B.S.N., Carbone, B., Mistri, T.K., Wilson, V., Tomlinson, S.R., Kaji, K., 2018. Mapping transcription factor occupancy using minimal numbers of cells in vitro and in vivo. Genome Res 28, 592–605. https://doi.org/10.1101/gr.227124.117**

Minor comments:
1) Add peak annotation in Figure 1C-D.

**This has been corrected.**

2) Please correct the color scale in Figure S1A-E-F and S2A so the white color aligns with the middle of the scale bar (i.e. value of 0,5 in figures S1A and S2A and of 0 in Figure S1E-F).

**This has been corrected.**

1: https://en.wikipedia.org/wiki/Pearson_correlation_coefficient
2: Mukaka MM. Statistics corner: A guide to appropriate use of correlation coefficient in medical research. Malawi Med J. 2012 Sep; 24(3):69-71
3: Hauke J., Kossowski T.. Comparison of Values of Pearson's and Spearman's Correlation Coefficients on the Same Sets of Data. Quaestiones Geographicae. 2011; 30(2):87-93.

*Reviewer #2: The authors addressed all of my concerns in the revision and I believe the manuscript is acceptable for publication now.

*Reviewer #3: My concerns have been addressed with the revised submission and the manuscript is suitable for publication.
